# Intensity modulated radiation therapy (IMRT) for the treatment of unicentric Castlemans disease: a case report and review of the use of radiotherapy in the literature

**DOI:** 10.2478/v10019-012-0008-0

**Published:** 2012-01-02

**Authors:** Chance Matthiesen, Rajeev Ramgopol, Jonathan Seavey, Salahuddin Ahmad, Terence Herman

**Affiliations:** Department of Radiation Oncology, University of Oklahoma Health Sciences Center, Oklahoma City, OK, USA

**Keywords:** Castleman’s disease, IMRT

## Abstract

**Background:**

Surgical resection is considered standard therapy for cases of resectable unicentric Castleman’s disease (UCD). Unresectable cases of UCD do not have a consensus regarding the optimal treatment approach, but have utilized steroids, observation, chemotherapy, and radiotherapy. Here we discuss a patient presentation of UCD treated with an advanced radiotherapy technique, IMRT.

**Case report:**

A 47 year old female was found to have an intra-thoracic posterior UCD and was determined not to be a good surgical candidate. She was referred for radiotherapy and was treated using IMRT to a total dose of 4320 cGy in 180 cGy fractions including a scheduled 10 day break. Following the break, the patient’s treatment was replanned at which the initial treatment volume was reduced by 50.9% for the duration of the treatment course. Radiation Therapy Oncology Group (RTOG) grade III pneumonitis developed which was managed medically. Neither disease progression nor late effects have occurred.

**Conclusions:**

The use of IMRT and planned treatment break was successful in the treatment of a case of UCD, and should be considered for other unresectable cases.

## Introduction

Castleman’s Disease (CD) was first described in 1954 and further characterized in 1956 by Benjamin Castleman when he reported on 13 cases of localized mediastinal lymphoid hyperplasia.^1^ CD, also known as angiofollicular lymph node hyperplasia or giant lymph node hyperplasia, is a rare disorder of unknown etiology. As originally described, CD was a localized disease limited to a single lymph node, which is now referred to as Unicentric Castleman’s Disease (UCD). In 1978, Gaba *et al.* recognized the multicentric Castleman’s disease manifestation (MCD).^2^ Little is understood regarding the pathogenesis of CD. An association was noted between human herpes virus 8 (HHV-8), human immunodeficiency virus (HIV), and MCD. This association has fueled theories as to the pathology of MCD, but has contributed little to the understanding of UCD or HHV-8 negative MCD.^3^–^5^

Clinically, UCD tends to be asymptomatic and is frequently diagnosed incidentally. MCD frequently presents with systemic manifestations at presentation such as fever, weight loss, diaphoresis, and fatigue. The disease progression of CD has been described as ranging from indolent to fulminant.^6^ Additionally, CD has been associated with increased risk of lymphoma, amyloidosis, renal insufficiency, and POEMS (polyneuropathy, organomegaly, endocrinopathy, monoclonal gammopathy, skin changes) syndrome which has sometimes guided treatment decisions.^6^,^7^

Standard treatment guidelines are lacking, and there is a paucity of evidence owing to the rarity and heterogeneity of the disease. Literature has been limited to mostly case reviews and a few small case-series. Surgery is considered standard therapy for resectable UCD with several case reports and retrospective series reporting excellent local control and cure rates.^8^–^10^ An alternative successful treatment approach for UCD has included the use of radiotherapy.^11^–^14^ Other treatment approaches have included steroids, observation, chemotherapy, and combinations of the above mentioned modalities.^13^,^15^ Unresectable cases of UCD do not have a consensus regarding the optimal treatment approach.^14^ The treatment for MCD is even less understood and is beyond the scope of this paper. This paper reviews and illustrates a case of UCD that was incidentally found and treated with primary radiotherapy, specifically intensity modulated radiation therapy (IMRT). Furthermore, we aim to review and summarize prior reports regarding the use of radiotherapy.

## Case report

### Patient presentation

**FIGURE 1:**
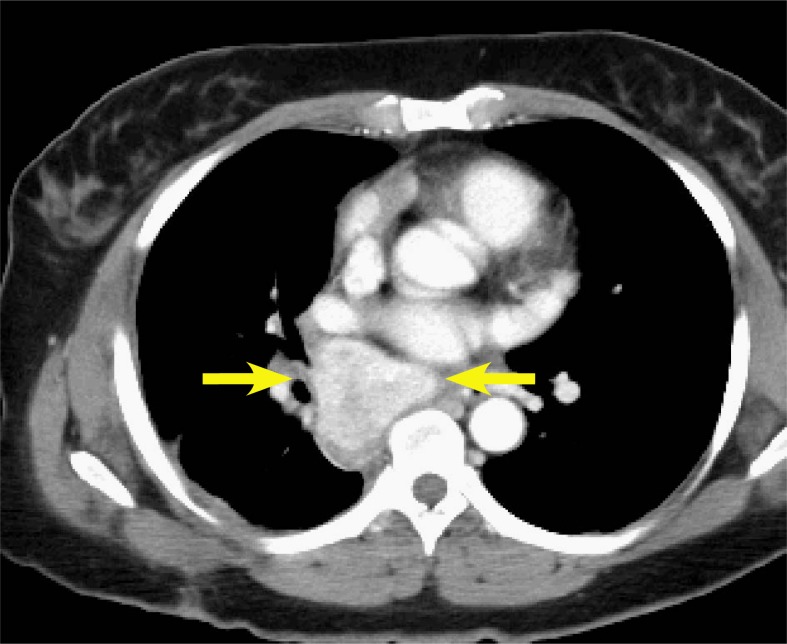
CT of patient at initial presentation with a mass noted in the posterior right mediastinum.

The patient is a 47 year old Native American female without significant past medical history or known CD risk factors who presented after being critically injured in a motor vehicle accident. During her trauma evaluation and stabilization, a posterior mediastinal mass was noted as shown in Figure 1. She was taken to the operating room for multiple internal injuries necessitating surgical repair. Visualization of the mass was noted but was not attended to at that time. After a several month recovery, she returned to the thoracic surgeon for follow-up and further workup of the mass. Biopsy was performed via mediastinoscopy and pathology results revealed angiofollicular lymph node hyperplasia, consistent with CD. CT and PET-CT scans were performed which showed the mass to be 5.5 cm x 4.6 cm with a peak SUV of 5.3. Due to the location of the mass and history of recent prior surgery, she was determined not to be a good candidate for complete surgical resection. She was then referred to consider radiotherapy treatment options.

### Treatment

**FIGURE 2:**
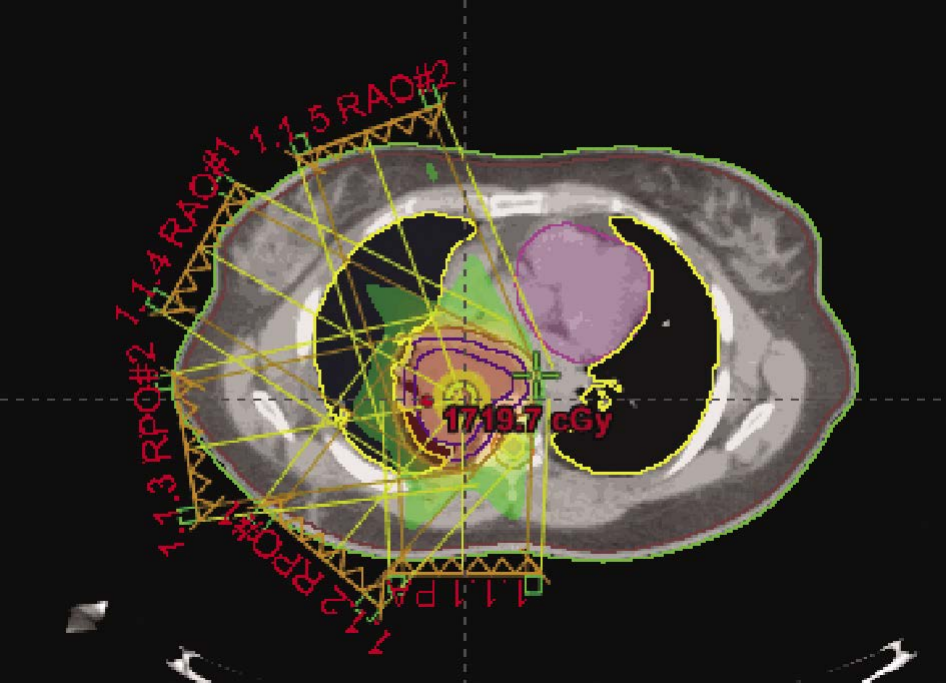
Illustration of the contoured mass and five field (1.1.1–1.1.5) coplanar beam arrangements for the IMRT treatment plan.

**FIGURE 3:**
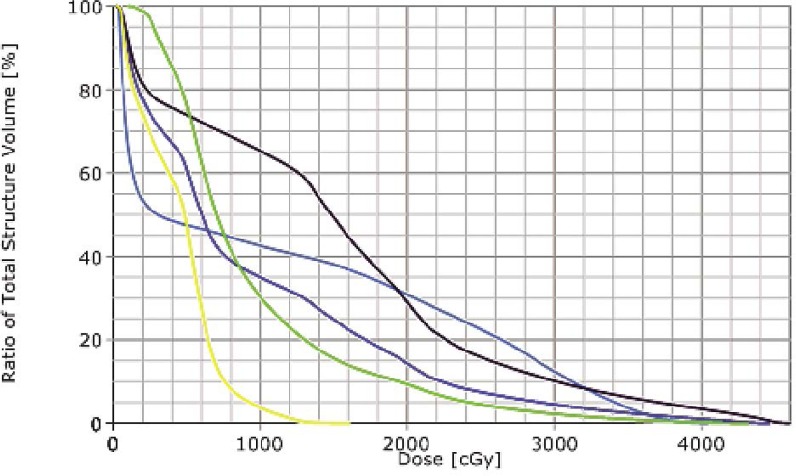
DVH of initial IMRT treatment plan. Line colors as follows: Orange - Spinal Cord, Pink - Heart, Yellow - Total Lung, Green - Right Lung, Blue - Left Lung.

**FIGURE 4:**
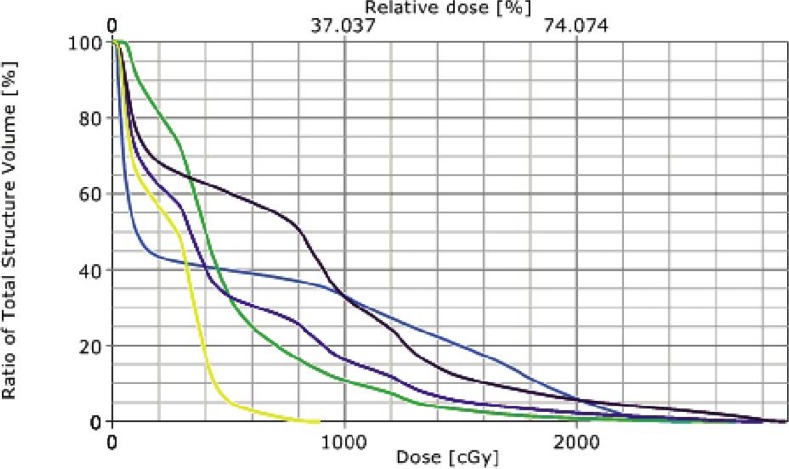
DVH of post treatment break IMRT treatment plan. Line colors as follows: Orange - Spinal Cord, Pink - Heart, Yellow - Total Lung, Green - Right Lung, Blue - Left Lung.

**FIGURE 5:**
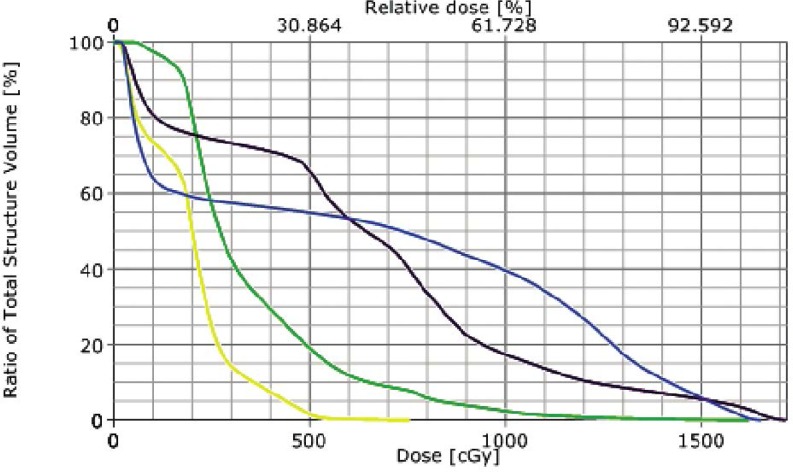
DVH of Cumulative IMRT treatment plans. Line colors as follows: Orange - Spinal Cord, Pink - Heart, Yellow - Total Lung, Green - Right Lung, Blue - Left Lung.

Treatment simulation was performed in the supine position and immobilization via a vac-lock. Treatment planning was performed on Eclipse External Beam Planning 7.5.51 (Varian Medical Systems, Palo Alto CA). Given the midline, posterior location of the mass, IMRT was utilized to reduce the dose gradient and toxicity to the surrounding normal tissues. The gross tumor volume (GTV) was contoured and expanded 5 mm to create the planning target volume (PTV). The patient was treated to a total dose of 4320 cGy. RT was delivered in nine initial once daily fractions of 180 cGy to a total dose of 1620. She was then placed on a 10 day break followed by re-simulation and treatment planning. She then completed the total 4320 cGy dose prescription without interruption in 180 cGy once daily fractions. Radiation therapy (RT) beam arrangements are shown in Figure 2. The initial PTV volume was 235.7 cm^3^ and the re-CT PTV following treatment break was reduced to 120 cm^3^, a reduction of approximately 50.9%. Cumulative dose volume constraints allowed the mean total lung dose to be limited to 961 cGy and the 20% volume to be limited to 1706 cGy. The heart was limited to a mean dose of 939 cGy, and the expanded cord mean to 1163 cGy. Dose volume histograms of each plan (pre and post treatment break) and a cumulative plan sum are shown in Figures 3, 4, and 5. A summary of mean and max dose statistics is shown in Table 1. No acute side effects were noted during treatment.

### Follow-up

**FIGURE 6:**
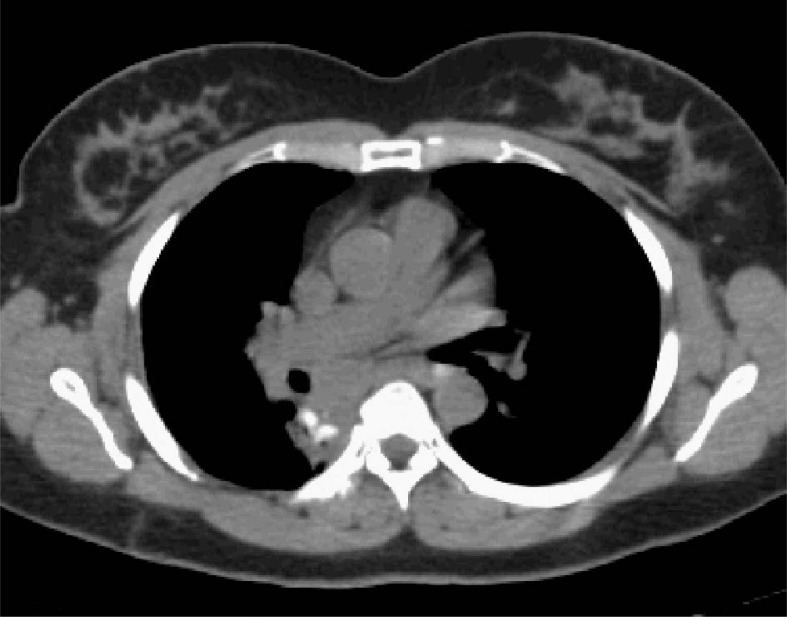
CT at four months post-RT. The mass is noted to be smaller.

At a follow-up of ten months, the patient has had no disease progression. She did develop RTOG grade III pneumonitis at three months follow-up which was managed by steroids. Follow-up PETCT (Figure 6) showed the mass to be stable at 3.9 cmx 5.1 cm and SUV values to be at background. No late effects have occurred.

## Discussion

Consensus suggests that the optimal treatment approach for patients presenting with UCD has been surgical resection, as cure rates following this approach are near 100%.^8^–^10^ The most well-known evidence for this is from a retrospective report of sixty-one patients with UCD treated with surgery and followed for twenty years.^16^ This report illustrated that complete resection offered the best chance of cure. Other reports have shown that partial resections can ameliorate constitutional manifestations in symptomatic patients; however, documented recurrences did occur more than nine years post resection so continued follow-up is necessary.^12^,^13^

The approach and treatment of unresectable UCD is not standardized, as methods including combinations of observation, steroids, surgery, chemotherapy, and radiotherapy in multiple settings have been utilized.^14^ Additional reports have commented on the use of radiotherapy.^11^–^14^,^17^,^18^ To the best of our knowledge, no previous reports have commented on the use of IMRT, which is now used routinely for diseases involving the thorax such as lymphoma and cancers of the lung and esophagus as well as for other localisations.^19^–^24^

In our report we illustrated the successful use of IMRT for a case of unresectable UCD. Previous reports have utilized cumulative doses ranging from 12 to 50 Gy^11^,^12^, with lower doses resulting in disease remission in some cases,^25^–^28^ but failure in others.^2^,^16^,^29^ A prior review of the literature^14^, showed no correlation between dose and response when using primary radiotherapy.

Neuhof and Debus reported on five patients treated with radiotherapy with doses ranging from 40–46 Gy.^11^ Four patients were alive at analysis and all four achieved disease stabilization, or a partial / complete response to radiotherapy. Treatment was well tolerated with the exception of one patient who developed acute grade III skin reactions, followed by stenosis of the trachea, esophagus, and bronchus. Treatment technique in this patient was 3D conformal planning.

Chronowski *et al.* reported on 21 CD patients.^12^ Twelve had UCD of which four were treated with radiotherapy. All four responded favorably to treatment, with three complete responses to radiotherapy and one partial response. At analysis, two were disease free and the other two had died of unrelated causes. Radiotherapy doses ranged from 39.6 to 40 Gy at 1.8 and 2 Gy fractionation. No comments of toxicity were reported.

De Vries *et al.* reported the use of neoadjuvant radiotherapy for a case of unresectable UCD.^14^ In their technique, they used a conventional approach for a lesion in the abdomen treated in 2 Gy fractions to 40 Gy. A 10 Gy intraoperative boost was applied at the time of surgery. No radiotherapy complications were reported. They concluded a neoadjuvant approach could be applied to other cases of UCD.

In our report, we describe the use of IMRT for UCD. To our knowledge, no previous reporting of this technique for CD is in the literature. IMRT has been well studied and known to be effective for diseases involving the chest to allow radiotherapy dose escalation with minimization of normal tissue toxicity.^20^,^21^,^30^,^31^ Additionally, we introduce the application of a treatment break during radiotherapy to allow for tumor size reduction. Given the lymphoid nature of CD, and the known apoptotic response of lymphoid tissues to radiotherapy^32^, a reduction of tumor size in CD could reasonably be expected. In our case, we treated the tumor mass to 1620 cGy, then allowed a for 10 day break followed by re-simulation and treatment planning. As a result, the initial PTV was reduced by approximately 50.9%. This PTV volume reduction resulted in further dose minimization to the normal lungs, esophagus, and spinal cord following IMRT treatment replan. Although a dosimetric comparison of radiotherapy techniques is not the intent of this discussion, it should be understood that the use of IMRT is more optimal versus 3D conformal techniques to achieve these results. Furthermore, it should be noted that IMRT can allow greater dose escalation for such tumors; though no evidence is available supporting such a decision. Other modern photon techniques which could be considered include arc therapy and tomotherapy, as in certain situations these techniques may be able to provide an improved normal tissue toxicity dose reduction or more rapid treatment administration. As proton therapy becomes more available, such patients and lesion locations may be most optimally treated by this method to benefit from the proton beam dose characteristics of the Bragg peak. Since unicentric CD often appears in younger patients^11^, methods and techniques to limit normal tissue dose should always be taken into consideration.

## Conclusions

The dosimetric advantages of IMRT make it a suitable treatment approach for unresectable UCD. A treatment break during therapy should also be considered depending upon lesion location and patient characteristics to further reduce treatment target volumes and improve the dose gradients to the normal tissue.

### Clinical practice points

The approach and treatment of unresectable UCD is not defined, as methods including combinations of surgery, chemotherapy, and radiotherapy in multiple settings have been utilized. Additional reports have commented on the successful use of radiotherapy. No previous reports have commented on the use of IMRT, which is now used routinely for diseases involving the thorax such as lymphoma and of the lung and esophagus. The dosimetric advantages of IMRT make it a suitable treatment approach for unresectable UCD. In our report, we highlighted the successful use of IMRT for an unresectable mediastinal mass in a younger patient with UCD. We also described the application of a planned treatment break followed by resimulation and replan of the treatment course. Such an approach allowed the planning treatment volume to be reduced by 50.9% which subsequently allowed greater normal tissue sparing and dose reduction. At follow-up, the patient has had a partial response to radiotherapy and disease stabilization. Therefore, we suggest that the use of IMRT should be considered for unresectable unicentric Castleman’s disease and the implementation of a treatment break should be considered.

## Figures and Tables

**Table 1:** Organs at risk and the mean and max dose values for each individual treatment plan and cumulative plan.

**Organ**	**Initial Plan Dose (cGy)**	**Replan Dose (cGy)**	**Cumulative Dose (cGy)**
Spinal Cord	Mean (672), Max(1656)	Mean (633), Max(2493)	Mean (1163), Max(4057)
Right Lung	Mean (638), Max(1717)	Mean (780), Max(2908)	Mean (1496), Max(4609)
Left Lung	Mean (196), Max(761)	Mean (246), Max(895)	Mean (447), Max(1617)
Total Lung	Mean (394), Max(1663)	Mean (504), Max(2813)	Mean (961), Max(4476)
Heart	Mean (355), Max(1622)	Mean (501), Max(2686)	Mean (939), Max(4335)
